# Effect of postoperative 660-nm low-level laser therapy on the radiographic crestal bone loss of fresh-socket dental implants

**DOI:** 10.34172/joddd.29923

**Published:** 2024-09-07

**Authors:** Alireza Sighari Deljavan, Hasan Momeni, Amirmansour Shirani

**Affiliations:** ^1^Department of Oral and Maxillofacial Surgery, Faculty of Dentistry, Islamic Azad University, Tabriz Branch, Tabriz, Iran; ^2^Department of Oral and Maxillofacial Surgery, Faculty of Dentistry, Islamic Azad University, Isfahan (Khorasgan) Branch, Isfahan, Iran; ^3^Department of Oral Science, Faculty of Dentistry, Islamic Azad University, Isfahan (Khorasgan) Branch, Isfahan, Iran

**Keywords:** Bone resorption, Dental implant, Diode laser, Periapical intraoral radiography

## Abstract

**Background.:**

Although the benefits of low-level laser therapy (LLLT) in soft tissue healing have been demonstrated, the effects of laser on bone have remained controversial. This study investigated the impact of postoperative 660-nm LLLT on the radiographic crestal bone loss of fresh-socket dental implants.

**Methods.:**

Thirty patients referred to the Department of Oral and Maxillofacial Surgery for tooth extraction and placement of fresh-socket implants were selected and assigned to two groups: laser (intervention) and no-laser (control) groups. Immediately after tooth extraction, the implant was inserted into the tooth socket. 660-nm LLLT was immediately started after surgery and was repeated three times per week for two weeks. Bone quantification at the implant site was assessed using periapical intraoral radiographs and computerized software immediately after surgery and after six months.

**Results.:**

This study showed a statistically significant difference in mean bone resorption between the mesial and distal aspects of the two groups, with lower bone resorption in the laser group compared to the no-laser group.

**Conclusion.:**

The results of this study suggest that LLLT can effectively reduce bone resorption in fresh-socket implant placement. This might indicate the positive effect of LLLT on bone resorption reduction.

## Introduction

 Immediate implant placement after tooth extraction (fresh-socket implant placement) is a good technique with aesthetically pleasing results; it can shorten the treatment process and reduce the number of referral sessions.^1^ However, in this technique, the risk of complications and implant failure is higher because it might be difficult to obtain sufficient primary stability.^2^ The most common problems associated with fresh socket implants are inadequate 3D implant position, lack of sufficient keratinized tissue in the place, gingival resorption, bone resorption, surgical trauma-induced implant failure, surgical site infection, premature loading, and anatomical constraints such as bone quality and quantity.^3^

 Stable blood clot formation and proper repair of epithelial tissue have been identified as essential factors in obtaining successful osseointegration. For this purpose, protective membranes are recommended. However, it has been shown that immediate implant placement in a newly extracted tooth socket, with or without membranes, cannot prevent resorption, which accounts for more than 50% on the buccal and 30% on the palatal sides.^4^ Tadi et al. calculated the initial stability and resolution of crestal bone in patients undergoing fresh-socket implant placement, reporting that mean marginal bone resorption in fresh-socket implants was 1.23 mm after six months.^5^ A great deal of information is available regarding the use of low-level lasers in recent years to improve the healing process of oral tissues. The idea that low-level lasers can be therapeutic, reduce pain, and improve tissue repair has been intensely discussed among scientists and physicians.^6^ Some studies have shown that low-level lasers can improve the healing process of skin, ligaments, nerves, bones, and cartilage in animal experiments. However, wound healing in humans depends on many factors.^7^ Other studies have reported conflicting results, proposing that low-level lasers and other monochromatic light sources are ineffective in improving tissue repair, and there are doubts about their beneficial therapeutic effects.^8^ The number of studies on the effect of laser on dental implant treatments has increased in recent years, and they have aimed at providing more patient comfort through the effects of laser on postoperative pain and edema, reduction and improvement of postoperative paresthesia, and peri-implantitis treatment.^9^ Pinheiro et al.^10^ investigated the effect of a low-level laser as a biostimulator on the osseointegration and bone healing process after implant placement in the tibia of dogs. This study showed that the laser could improve the bone repair process at the interface between tissue and implant in the early stages of wound healing.

 In recent years, numerous methods have been used to improve the quality of the fresh-socket implantation technique and reduce its complications, such as reduced crestal bone resorption and bone wall surrounding the implant. To date, however, no study has investigated the effect of low-level laser therapy (LLLT) on crestal bone resorption in fresh-socket implants. Therefore, this study investigated the effects of LLLT on enhancing the quality of treatment and reducing postoperative complications so that LLLT can be included in the standard fresh-socket implant technique.

## Methods

 This double-blind, randomized clinical trial was performed at the Department of Oral and Maxillofacial Surgery, Islamic Azad University of Isfahan, between January 2019 and July 2019. In one month, 156 patients were screened during the routine examination to match the inclusion criteria:

- Individuals referred to the Department of Oral and Maxillofacial Surgery for tooth extraction and implant placement, with no inflammation or gingivitis
- Healthy adults at least 18 years old
- Sufficient bone density to receive the implant with no need for bone augmentation and no history of tooth extraction during the selected six months
- Having at least 6 mm of buccolingual ridge width at the site of implant placement for inserting an implant measuring at least 4 mm in diameter in the ideal position

###  Exclusion criteria

- Systemic: pregnancy or lactation, systemic diseases affecting osseointegration, using anticoagulants, systemic glucocorticoid therapy, history of radiotherapy in the craniofacial region in the last 12 months, smoking more than 10 cigarettes per day, oral cancer, history of seizures
- Local: Acute oral infection, untreated or uncontrolled periodontal disease

The study procedure and its alternatives and the probable risks and benefits of the low-level laser treatment were explained to the patients, and written informed consent was taken. The study design, which was under the Helsinki Declaration of Human Rights, was submitted to and approved by the Committee for Ethics in Research on Humans at the Islamic Azad University of Isfahan (Ref number: IR.IAU.KHUISF.REC.1397.072).

 According to a previous study,^5^ the mean marginal bone resorption in fresh-socket implants was 1.23 with a standard deviation of 0.6 mm after six months. Therefore, by considering the expected reduction in bone resorption to be approximately 0.5 mm with low-level laser, α = 0.05 and 80% power, the sample size for both intervention and control groups was 13, which was increased to 15 to improve the validity of the study and compensate for probable lost to follow-up cases or failure of implant treatment during the study period.

 Thirty patients were randomly divided into laser (intervention) and no-laser (control) groups. A random allocation list was generated using randomization software. Each patient could provide an area for implant treatment. In the examinations, the operator could freely choose the treatment area. The operator was not blinded to the treatment because of the different manipulation techniques implemented for the studied groups. All other contributors to the study were blinded to the generation and implementation of the treatment assignment.

 Demographic data and patients’ history were collected using a form. Panoramic and CBCT radiographs (if needed) were requested to select patients.

**Figure 1:**
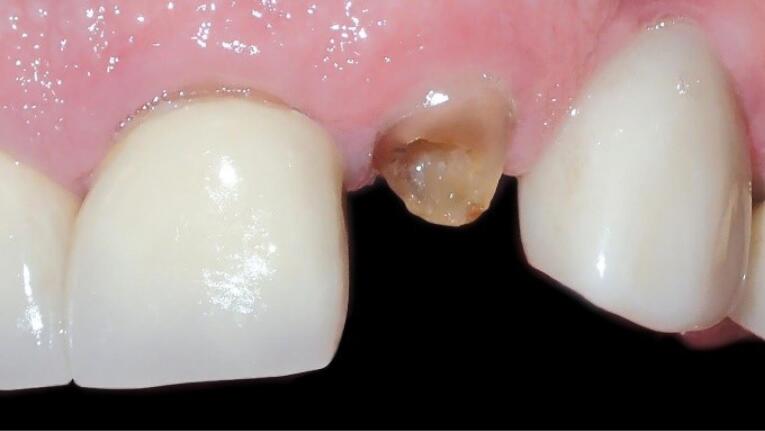
Preoperative intraoral view

**Figure 2:**
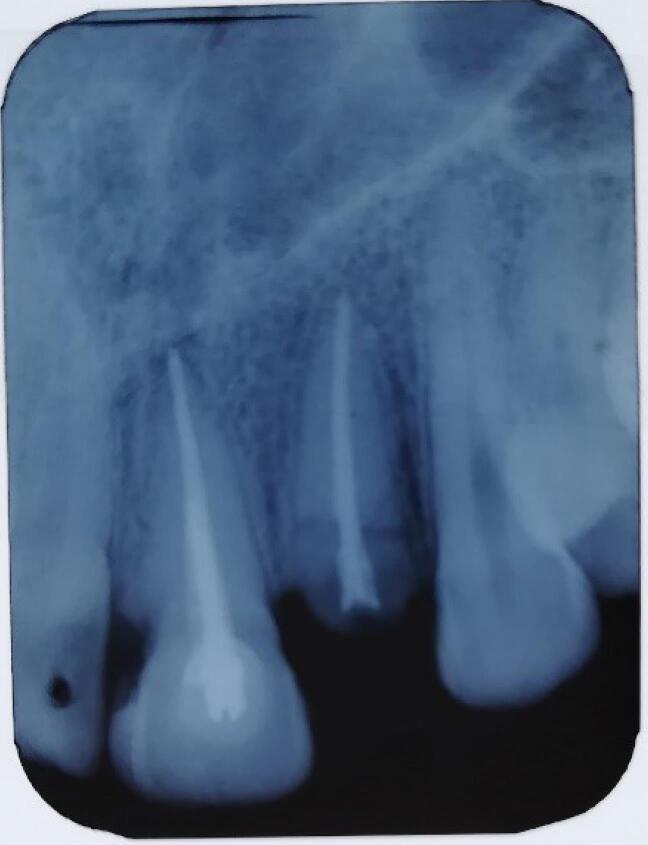
Preoperative intraoral periapical radiograph

**Figure 3:**
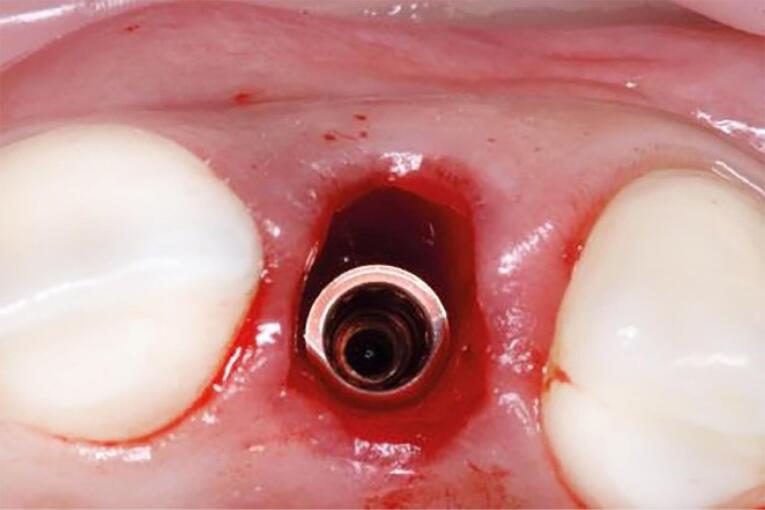
Implants placed in fresh extraction sockets

 One oral and maxillofacial surgeon with ten years of experience (the operator) performed all the surgical procedures. In both groups, after selecting the patients based on eligibility criteria (Figures 1 and 2), local anesthesia was performed by injecting 2% lidocaine (with 1:80 000 adrenaline). After cutting the crestal, elevating a mucoperiosteal flap, and extracting the tooth without trauma, the bone-level implant recipient site was prepared under cooling with a physiological serum solution according to the manufacturer’s protocol (Dio, Seoul, Korea). For all implants, a speed of 15 rpm with a torque of 35-40 Ncm was used. The diameter of the implants was chosen so that at least 1 mm of bone remained on both buccal and palatal aspects after implant placement. For vertical positioning, the implant was also positioned at the level of the buccal bone crest (Figure 3). The implant was placed, and the area was sutured. The sutures were removed seven days after surgery. After the surgery, all the patients received amoxicillin (1.5 g) or clindamycin (1.8 g) daily for three days and a non-steroidal anti-inflammatory drug for pain relief, in association with mouthwash. The patients also received advice regarding oral hygiene. No temporary dentures were placed during the 6-month follow-up period.

**Figure 4:**
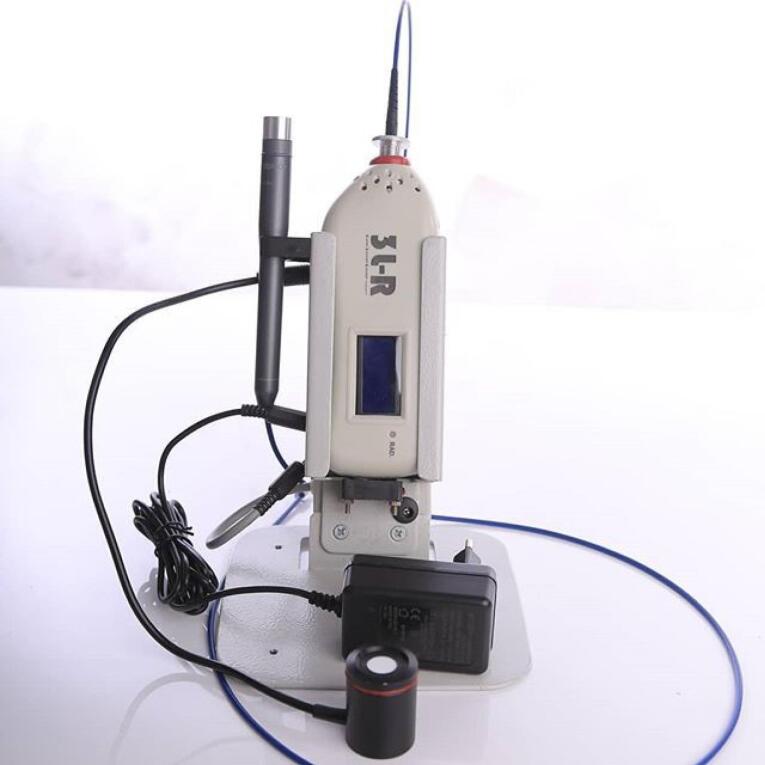
Low-level 660-nm diode laser (Hamerz, Iran)

**Figure 5:**
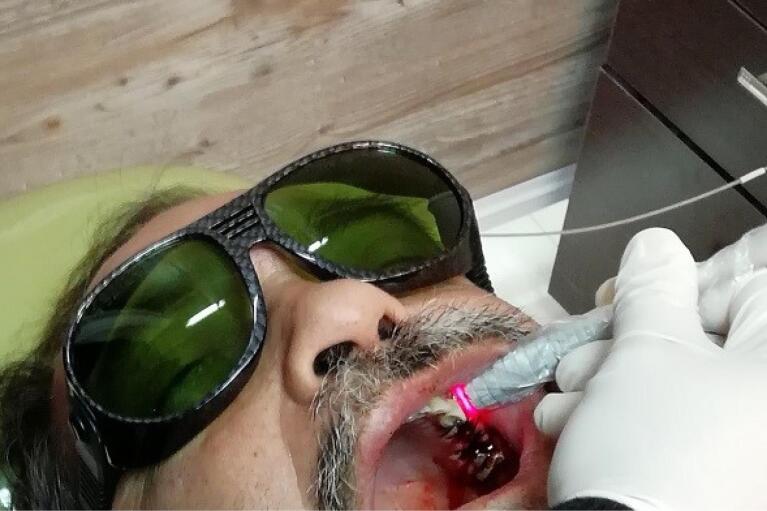
Postoperative low-level laser therapy

 A low-level 660-nm diode laser (Hamerz, Iran) (Figure 4) was delivered (6.26 J/cm^2^, 0 Hz, 400 mW) to the surrounding tissues of the implant along its longitudinal axis in this study.^11^ Low-level laser treatment (LLLT) was undertaken immediately after the surgery and repeated three times per week for two weeks (Figure 5).^12^ Output power was checked before working with the power meter. No laser was used in the control group.

 Bone quantification was performed at the implant site using periapical intraoral radiography with a parallel technique using a film holder to minimize photo distortion (Rinn XCP, Dentsply). Radiographic evaluation was performed twice, immediately after implant placement and after six months. The position of the film was also recorded to ensure the reproducibility of the graphics during postoperative x-ray examinations by the Coltene Speedex system (Coltene, Switzerland) so that the same positional graphics could be obtained six months later. Periapical radiographs were obtained using a computerized scanner with specifications as follows: 600 dp and 250 gray scales scanned by Image Tool software to measure distances in digital photographs. Corresponding distances were measured while using the software.

**Figure 6:**
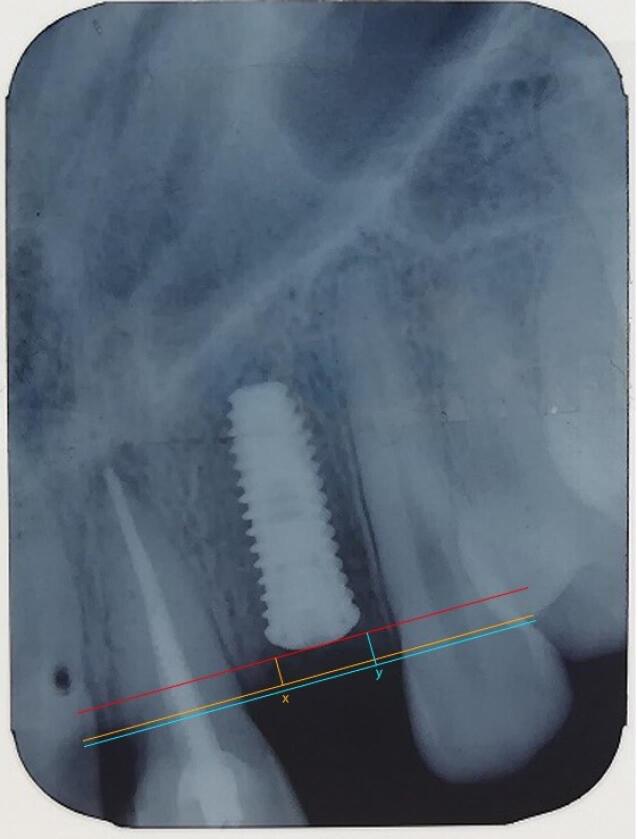
Measurement of crestal bone level from the reference line

**Figure 7:**
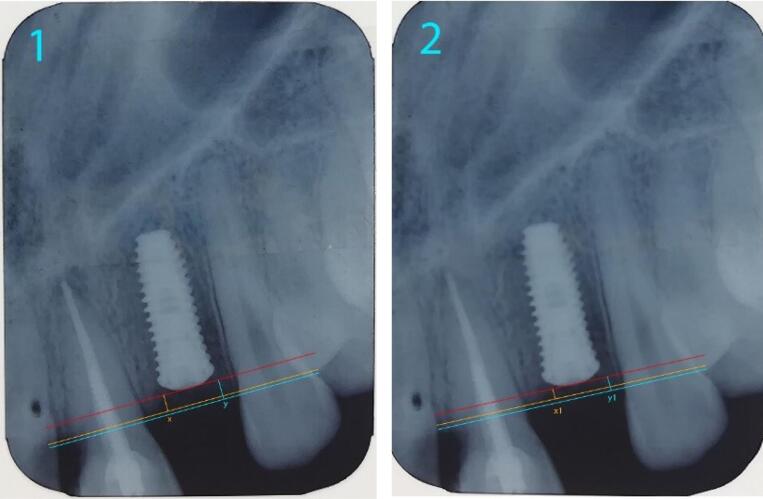
Comparison of crestal bone resorption (at 0 month and 6 months)

 Since the length of the implant used was identified, it was possible to use this length to calibrate the image in computer software. The coronal implant surface was considered the reference line. Two lines were drawn parallel to this line from the crestal bone ridge between the implant and adjacent teeth on both the mesial and distal sides of the implant, and the distance between the two lines and the reference line was measured (Figure 6). By decreasing the bone measurement immediately after implantation and six months later, the amount of bone resorption was achieved on both the mesial and distal sides of the implant (Figure 7).^5,13^

 The amount of bone resorption was calculated by two experienced oral and maxillofacial radiologists (not the operator) blinded to the technique, using the software to identify the smallest pixel identifiable from the bone, according to the patient’s radiographs. In case of disagreement, the examination of a third examiner was recorded as the treatment outcome.

###  Statistical analysis

 Patients̓ records and radiographic results were entered into SPSS 15 software. Descriptive statistics of means, standard deviations, and mean differences were used to describe the data. To analyze the data and investigate the difference in bone resorption size in the intervention and control groups, the normality of the data was evaluated using the Kolmogorov-Smirnov test. Then, the data were analyzed using the independent-samples t-test and Mann-Whitney U test, respectively. *P* < 0.05 was considered statistically significant for all analyses.

## Results

**Figure 8:**
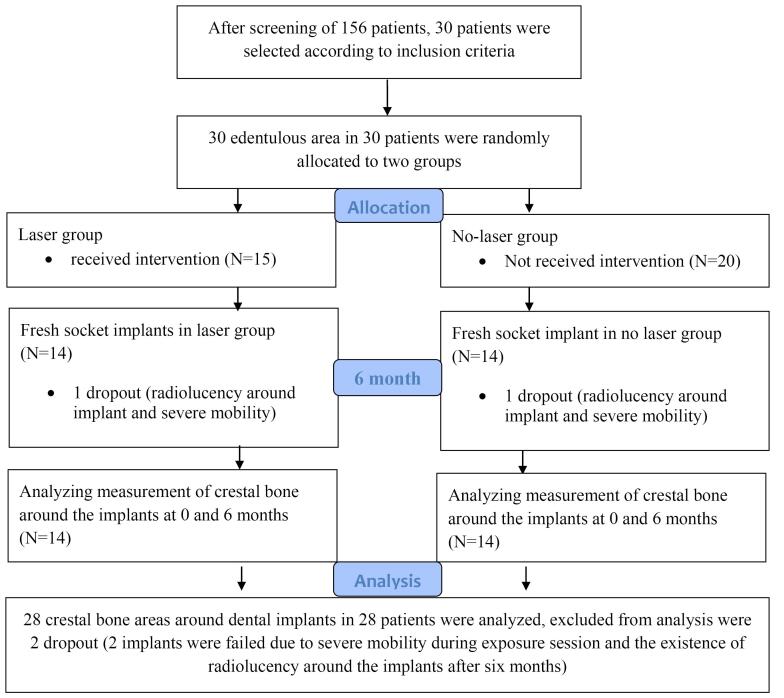
CONSORT diagram

Figure 8 presents the CONSORT diagram. Thirty non-restorable teeth were evaluated in 30 individuals (20 males and 10 females; mean age = 46.72).

Table 1 reports information on the implant specification used for participants. The treated site and implant data for the studied patients are presented in Supplementary file 1. Supplementary file 2 provides the studied patients’ bone level and loss data.

**Table 1:** Frequency distribution of implant lengths, diameters, and sites of implant placement in the studied patients

**Implant data**		**Frequency of placement**	**Percent**
Implant length	10 mm	20	66.7
	11.5 mm	10	33.3
Implant diameter	4 mm	18	60.0
	4.5 mm	12	40.0
Implant location	Maxilla	20	66.7
	Mandible	10	33.3

 The agreement between the examiners at baseline and 6-month follow-up was excellent (Baseline kappa = 0.92, *P* < 0.001 and final follow-up kappa = 0.94, *P* < 0.001).

 In the follow-up session after six months, one implant from each group had radiolucency around the insertion area, as evidenced by a periapical radiograph. After exposure, these two implants had severe mobility and were excluded from the study.

 As shown in Table 2, the mean mesial bone resorption was 0.552 mm in the laser therapy group, with 1.40 mm in the control group. The mean difference in mesial bone resorption was significant between these two groups (laser therapy and no-laser therapy) (*P* < 0.05).

**Table 2:** Comparison of the mean mesial bone resorption between the two groups

**Mesial bone resorption**	**Independent-samples t-test**					
	**N**	**Mean**	**SD**	**t**	**df**	***P*** ** value**
Laser therapy group	15	0.527	0.18	-5.489	26	0.001
No-laser therapy group	15	1.140	0.38			

 As shown in Table 3, the mean distal bone resorption was 0.559 mm in the laser therapy group, with 1.024 mm in the control group. The mean difference in distal bone resorption was significant between these two groups (laser therapy and no-laser therapy) (*P* < 0.05).

**Table 3:** Comparison of the mean distal bone resorptions between these two groups

**Distal bone resorption**	**Independent-samples t-test**					
	**N**	**Mean**	**SD**	**t**	**df**	***P*** ** value**
Laser therapy group	15	0.591	0.22	−4.288	26	0.001
No-laser therapy group	15	1.024	0.30			

## Discussion

 Immediate implant placement in a newly extracted tooth socket (fresh socket implant) has many advantages, including reduced overall treatment time and the number of surgical procedures, implantation in a more ideal position, better preservation of the height and contour of the soft tissue, and better osseointegration conditions due to its potential for healing of the newly extracted tooth socket.^14^

 Osseointegration is an essential prerequisite for the long-term prognosis of dental implants. Therefore, adjuvant chemical, biological, and biophysical therapies have been extensively studied to improve and accelerate bone and implant interfaces.^15^ This study was performed on the effect of low-level laser after the surgery using a low-level 660-nm diode laser with 100-mW output power and a total radiation dose of 6.26 J/cm^2^ for each implant to evaluate the rate of mesial and distal crestal bone resorption through standard periapical radiography 6 months after the surgery, prior to implant loading. This study aimed to evaluate the effect of LLLT on reducing bone resorption after dental implant placement.

 Tissue repair is a complex process involving local and systemic organic activities. Fibroblasts are a group of cells directly contributing to this mechanism. The use of lasers in the healing process has a broad role in inducing topical and systemic regenerative, anti-inflammatory, and analgesic effects.^16,17^ These effects have been demonstrated both in vitro and in vivo, especially in those studies focusing on increased local microcirculation, lymphatic system activity, proliferation of epithelial cells and osteoblasts, and increased collagen synthesis by osteoblasts.^18,19^ Pinheiro et al.^10^ suggested that although the benefits of laser in soft tissue repair have been demonstrated, there are still controversies on the effects of laser on bone, and conflicting findings have been reported.

 Long-term preservation of the height of the crestal bone around the osseointegrated implant is often considered an indicator of early success for different implant systems. Bone radiographic examination is an important and valuable indicator for identifying the health and stability of the area around the implant. A decrease in the level of the crestal bone indicates that the implant is losing its bone anchorage. Pathologic changes in follow-up sessions always begin to appear around the neck of the implant.^20^ Jung et al^21^ found that more than 50% of total bone resorption occurring in the first 12 months after implantation is within the first 3 months. Rapid primary bone resorption may be the result of damage to the periosteum, surgical trauma, receptor site preparation, and accumulated stress during implant placement.^22^ Wider resorption of the crestal bone during one year of implant placement occurs for many reasons, including surgical trauma, occlusal overload, peri-implantitis, microgaps, re-establishment of the biological width, and crestal bone pattern.^20,23^

 Many factors affect the survival and success of the implant. Studies have shown that subtle changes in the shape, length, and width of endosseous implants can influence success rates.^24^

 Radiographs are an important tool for assessing the bone level and evaluating stress focused around the implant, thus avoiding excessive alveolar bone resorption. Several radiographs are used to evaluate the implant recipient site, including intraoral periapical radiography, panoramic radiography, computed tomography, and similar modalities.^25^ Small changes in the level of the crestal bone emphasize the need for accurate and repeatable techniques for radiographic examination of bone height.^26^

 Standardized periapical radiographs are very useful for the long-term evaluation of peri-implant bone resorption.^27^ There are some problems regarding the use of panoramic radiographs, such as irreversibility, lack of sharpness, distortion of images, and superimposition of bone structures of the vertebrae.^28^ Also, different magnifications in each area, reduced resolution, and lack of standard radiation geometry indicate a risk of loss of measurement accuracy.^27^ The ability to image a large area by this technique can be useful in the initial treatment plan, which often involves examining the distance of the alveolar crest to the mandibular canal, the mental foramen in the mandible, and the floor of the maxillary sinus, nasal cavity, and incisal canal in the maxilla.^29^ Oblique cephalometric techniques require specialized radiographic equipment.^30^ According to Duckworth et al,^31^ periapical radiographs have minimal distortion when they are well-angulated by standardized radiation geometry. In addition, the exposure dose of periapical radiographs is much lower than in other modalities. Due to the sharpness and resolution of images obtained from standard periapical radiographs by the long-cone paralleling technique, the values measured by these radiographs are more accurate than the others.^27^ In addition, standardized periapical radiographs have the highest reproducibility and reliability regarding linear distance measurement, while the reproducibility of the radiographs obtained by the intraoral bisecting angle technique is poor.^28^ Therefore, parallel periapical radiographs are suitable for long-term bone resorption studies around the implant.^27^

 Different methods have been used to evaluate bone height in the implant area, such as counting threads of screw-type implants, measurements using computer software, and measurements using a magnifying glass or a digital sliding gauge.^28^

 Thus, the present study evaluated the changes in peri-implant crestal bone levels using standardized periapical radiographs in both laser and non-laser groups. These radiographs were obtained by the parallel technique using radiographic film holders (Rinn XCP; Dentsply).^27,32-34^ The patient-specific occlusal jig was made using a putty molding material, which was attached to the film holder during shooting, and the patient was asked to bite it. This jig was maintained for later visits to standardize the location of the film and cone angulation.^33^ One radiograph was obtained immediately after implant placement, and the others were taken six after the placement before prosthetic loading. To obtain repeatable data, it is important to define reference points in the images. The most coronal point of the implant was considered a reference because it is permanently visible and easily identified in radiographs.^35,36^ The highest crestal bone level between the tooth and the implant in the mesial and distal aspects was considered the crestal bone level in the mesial and distal sides. Measurements were performed by computer software^36^ immediately after the surgery and six months after the surgery in the mesial and distal sides of implants by vertical lines from the reference line to the crestal bone level on both sides. All radiographs should have a clear image of the implant and surrounding bone. The clear and visible threads of implants indicated that the central x-ray beam had been directed perpendicular to the object and film.^37,38^

 As shown in Table 2, the mean crestal bone resorption observed on the mesial side of the implant six months after implant placement was 0.527 mm in the laser therapy group and 1.140 mm in the control group. The mean difference of crestal bone resorption on the mesial side was significant between the two groups at 0.05 level (*P* < 0.05 was considered statistically significant). However, the mean crestal bone resorption on the distal side was 0.591 mm in the laser therapy group and 1.024 mm in the control group. The mean difference between the mean crestal bone resorption on the distal side was significant at 0.05 level for these two groups. Stein et al^39^ showed that helium-neon laser irradiation improves proliferation and evolution of human osteoblasts. Some studies have also shown the positive effect of laser irradiation on wound healing and collagen synthesis.^40,41^ In addition, low-level lasers have been shown to modulate inflammation, stimulate cell proliferation, and induce angiogenesis. The results of this study are consistent with those of a study by Singh et al,^35^ in which the mean bone resorption 6 months after implant placement was 0.6 mm in the mesial and 0.9 mm in the distal implant. Similarly, Behneke et al^42^ showed an average bone resorption of 0.8 mm between implant placement and prosthetic restoration. In contrast to these studies, a study by Johanson Ekfeldt^43^ showed an average bone resorption of 0.4 mm in the first year, followed by 0.1 mm annual bone resorption around the implants. Adell et al^22^ showed that the average bone resorption of osseointegrated implants was 1.5 mm in the first year. Zarb and Cox^44^ reported 1.6 mm of bone resorption in the first year, followed by 0.13 mm in each subsequent year. Bryant and Zarb^45^ showed no difference in proximal crestal bone resorption of dental implants in young and old subjects and reported a mean bone resorption of 1.4 mm in one year. According to the results of some studies, marginal bone resorption should not be more than 1.5 mm in the first year (osteointegration period) and 0.1 mm in subsequent years (follow-up period).^45,46^ Zarb and Smith suggested that alveolar bone resorption of < 0.2 mm per year after the first year is an indicator of implant success.^47^

 Therefore, most previous studies reported alveolar bone resorption of approximately 1.2 mm during the first year, followed by a constant mean of 0.1 mm for annual resorption. However, the most active phase of bone resorption during the first few months has not been extensively studied. Thus, in the current study, initial changes in the level of the crestal bone around dental implants were evaluated by standard intraoral radiographs within six months of implant placement before prosthetic loading in both laser therapy and no-laser therapy groups, and during the study period, the amount of bone resorption around the implant decreased significantly as a result of laser therapy.

 Differences in mean bone resorption reported by different authors can be attributed to different implant designs, surgeon’s experience, the number of implants studied, oral hygiene status, the time elapsed since implant placement, bone quality of the recipient site, and different measures used to evaluate the implant treatment methods. Rapid bone resorption in the first months after implantation might be caused by the lack of loading of the implants. Therefore, the absence of physiological stimulation and remodeling activities, independent of loading changes occurring right after the implant placement, can be effective in this bone resorption.

 Pham et al^48^ reported more significant crestal bone resorption before implant functional loading than when the prosthesis was attached. Jung et al.^21^ showed that over 50% of total bone resorption recorded in the first year occurs within the first three months. Several researchers have suggested that crestal bone resorption around the dental implant may be a normal occurrence because when adequate mucosal coverage is formed by the epithelial and connective tissues surrounding the implant, this resorption eventually becomes stable; however, other researchers suggested that crestal bone resorption might be the result of surgical trauma during implant insertion, removal of the periosteum, and implant osteotomy preparation. Bone resorption occurring during the first few months after the surgery might be attributable to bacterial invasion, re-establishment of biologic width, and factors that result in stress accumulation in the crestal area. Bacterial induction is the primary reason for bone resorption around the normal tooth. Occlusal trauma may accelerate the process, but trauma alone is not an influential factor. Peri-implant gingival sulcus in partial toothless patients has shown similar bacterial flora compared to normal teeth, leading to a reasonable assumption that bacteria primarily cause rapid bone resorption around the implant and that occlusal factors play a contributing or accelerating role. Poor oral health has been reported to accelerate bone resorption observed around endosseous implants.

 Since the number of samples in this study was relatively low, further studies with larger sample sizes are recommended.

## Conclusion

 Crestal bone resorption on the mesial and distal sides of fresh-socket implants was lower after six months in the laser treatment group compared to the non-laser treatment group, indicating a positive effect of LLLT on reducing bone resorption. Therefore, the results of this study showed that low-level 660-nm diode laser positively impacts crestal bone resorption in fresh-socket implants.

 Thus, some crestal bone resorption is unavoidable after the surgery; however, because of the success and durability of the implant, efforts should always be made to reduce this resorption.

## Acknowledgments

 The authors would like to thank the staff at the Departments of Oral and Maxillofacial Surgery and Oral and Maxillofacial Radiology for their assistance and the patients for participating in the study. The authors declared that they had no conflict of interest.

## Competing Interests

 The authors declare no competing interests with regard to the authorship and publication of this article.

## Ethical Approval

 The present study was approved by the Committee for Ethics in Research on Humans at the Islamic Azad University of Isfahan (Ref number: IR.IAU.KHUISF.REC.1397.072).

## Supplementary Files

Supplementary file 1. The treated site and implant data for the studied patients.

Supplementary file 2. Bone level and bone loss data for the studied patients.
