# Supplementary material for: Effect of postoperative 660-nm low-level laser therapy on the radiographic crestal bone loss of fresh-socket dental implants
Source: J Dent Res Dent Clin Dent Prospects. 2024 Sep 7;18(3):210–8. doi: 10.34172/joddd.29923 (PMC11459084; doi:10.34172/joddd.29923)
Supplement: Supplementary file 1 — The treated site and implant data for the studied patients. [file joddd-18-210-s001.pdf]

Sighari Deljavan et al, *J Dent Res Dent Clin Dent Prospects,* 2024, 18(3), 210-S1
doi: 10.34172/joddd.29923
https://joddd.tbzmed.ac.ir

Supplementary file 1. The treated site and implant data for the studied patients

Subject no.	Implant Length (mm)	Implant Diameter (mm)	Implant Location	Gender	Laser Therapy
S1	10	4	24	Male	Yes
S2	10	4	15	Male	Yes
S3	11.5	4	11	Female	Yes
S4	10	4.5	22	Male	Yes
S5	11.5	4	12	Male	Yes
S6	11.5	4	34	Female	Yes
S7	10	4.5	46	Male	Yes
S8	10	4.5	21	Female	Yes
S9	10	4	12	Male	Yes
S10	10	4.5	22	Female	Yes
S11	10	4	25	Male	Yes
S12	11.5	4.5	16	Male	Yes
S13	11.5	4	36	Male	Yes
S14	10	4	45	Female	Yes
S15	10	4	47	Female	Yes
S16	10	4	26	Male	No
S17	11.5	4.5	25	Male	No
S18	10	4.5	15	Female	No
S19	11.5	4	14	Male	No
S20	10	4.5	16	Female	No
S21	10	4	36	Male	No
S22	10	4.5	34	Male	No
S23	10	4.5	44	Male	No
S24	11.5	4.5	22	Male	No
S25	11.5	4	11	Female	No
S26	10	4	26	Male	No
S27	10	4	45	Female	No
S28	10	4.5	36	Male	No
S29	10	4	23	Male	No
S30	11.5	4	16	Male	No
