# Supplementary material for: Effect of postoperative 660-nm low-level laser therapy on the radiographic crestal bone loss of fresh-socket dental implants
Source: J Dent Res Dent Clin Dent Prospects. 2024 Sep 7;18(3):210–8. doi: 10.34172/joddd.29923 (PMC11459084; doi:10.34172/joddd.29923)
Supplement: Supplementary file 2 — Bone level and bone loss data for the studied patients. [file joddd-18-210-s002.pdf]

Sighari Deljavan et al, *J Dent Res Dent Clin Dent Prospects,* 2024, 18(3), 210-S2
doi: 10.34172/joddd.29923
https://joddd.tbzmed.ac.ir

Supplementary file 2. Bone level and bone loss data for the studied patients

Subject no.	Bone level from reference line (top surface of the implant) (average values) (mm)				Bone loss		Laser Therapy
	0 month		4 month				
	Mesial	Distal	Mesial	Distal	Mesial	Distal	
S1	3.43	2.98	2.86	2.37	0.57	0.61	Yes
S2	1.83	1.77	1.02	1.14	0.81	0.63	Yes
S3	2.22	2.60	1.73	2.22	0.49	0.38	Yes
S4	1.92	1.95	1.52	1.53	0.40	0.42	Yes
S5	1.70	1.98	1.40	1.37	0.30	0.61	Yes
S6	2.74	2.38	2.51	2.14	0.23	0.37	Yes
S7	3.12	3.75	2.62	2.60	0.50	1.15	Yes
S8	2.44	2.84	2.11	2.23	0.33	0.61	Yes
S9	3.86	3.54	3.42	3.08	0.44	0.46	Yes
S10	2.12	2.35	1.60	1.81	0.52	0.54	Yes
S11	3.18	3.30	2.42	2.38	0.76	0.92	Yes
S12	1.69	1.34	1.11	0.97	0.58	0.37	Yes
S13	2.78	2.69	2.06	2.16	0.72	0.53	Yes
S14	2.75	2.56	2.02	1.88	0.73	0.68	Yes
S15	1.54	1.63	Fail	Fail	Fail	Fail	Yes
S16	2.86	1.80	1.48	1.04	1.38	0.76	No
S17	1.80	1.69	0.74	0.85	1.06	0.84	No
S18	2.33	2.10	1.19	0.68	1.14	1.42	No
S19	3.46	3.12	1.36	1.43	1.90	1.69	No
S20	2.13	1.96	0.89	0.94	1.24	1.02	No
S21	3.87	3.56	2.23	2.45	1.64	1.11	No
S22	1.45	1.34	0.42	0.25	1.03	1.09	No
S23	2.11	2.20	1.28	1.42	0.83	0.78	No
S24	1.70	1.98	1.30	1.47	0.40	0.51	No
S25	2.14	2.31	1.28	1.28	0.86	1.03	No
S26	2.54	2.42	1.05	1.10	1.49	1.32	No
S27	1.90	1.79	0.78	0.91	1.12	0.88	No
S28	2.31	2.18	1.42	1.12	0.89	1.06	No
S29	1.31	1.24	0.33	0.45	0.98	0.79	No
S30	2.31	2.18	Fail	Fail	Fail	Fail	No
